# Antiangiogenics in Malignant Granular Cell Tumors: Review of the Literature

**DOI:** 10.3390/cancers15215187

**Published:** 2023-10-28

**Authors:** Carlos Torrado, Melisa Camaño, Nadia Hindi, Justo Ortega, Alberto R. Sevillano, Gema Civantos, David S. Moura, Alessandra Dimino, Javier Martín-Broto

**Affiliations:** 1 Medical Oncology Department, University Hospital Virgen del Rocío, 41013 Sevilla, Spain; ctorradomartin@gmail.com; 2 Medical Oncology Department, National Cancer Institute, 11600 Montevideo, Uruguay; ivacatto@yahoo.com; 3 Instituto de Investigacion Sanitaria Fundacion Jimenez Diaz (IIS/FJD), 28015 Madrid, Spain; nhindi@atbsarc.org (N.H.); jortega@atbsarc.org (J.O.); asevillano@atbsarc.org (A.R.S.); dmoura@atbsarc.org (D.S.M.); 4 Medical Oncology Department, Fundación Jimenez Diaz University Hospital, 28040 Madrid, Spain; 5 General de Villalba University Hospital, 28400 Madrid, Spain; 6 Pathology Department, Hospital Virgen del Rocío, 41013 Sevilla, Spain; gema.civantos.sspa@juntadeandalucia.es; 7 Department of Surgical, Oncological and Oral Sciences, Section of Medical Oncology, University of Palermo, 90127 Palermo, Italy; alessandradimino@gmail.com

**Keywords:** malignant granular cell tumors, MGT, granular cell tumors, Pazopanib, GCT, target therapy

## Abstract

**Simple Summary:**

Granular cell tumor (GCT) constitutes an ultra-rare subtype of soft tissue sarcoma (STS) that can exhibit aggressive behavior and limited survival in the metastatic setting. For metastatic GCTs, the therapeutic options are limited, as this tumor is relatively chemo-resistant. This review synthetizes the growing evidence for the relevant pazopanib benefit in advanced GCTs, while describing some insights on the pathology and the biology of these tumors. The data collected in this review suggest that pazopanib is substantially active in advanced GCTs in terms of dimensional responses. Pazopanib should be considered as a preferable treatment option for patients diagnosed with advanced GCTs.

**Abstract:**

Granular cell tumors (GCT) represent 0.5% of all soft tissue sarcomas (STS), and when metastatic, they exhibit aggressive behavior and determine limited survival. Metastatic GCTs are relatively chemo-resistant; however, there is growing evidence of the benefit of using pazopanib and other targeted therapies in this histology. This is a review of the role of pazopanib and other targeted therapies in the treatment of GCTs, along with some insights on pathology and molecular biology described in GCTs. From 256 articles found in our search, 10 case-report articles met the inclusion criteria. Pazopanib was the most employed systemic therapy. The median reported time on therapy with pazopanib was seven months. Eight out of ten patients (80%) experienced disease control with pazopanib, while four out of ten (40%) patients achieved an objective RECIST response. Molecular studies suggested that antitumoral effects of pazopanib in GCT might be due to a loss-of-function of *ATP6AP1/2* genes which consequently enhance signaling through several molecular pathways, such as SFKs, STAT5a/b, and PDGFR-β. Other reported targeted therapies for malignant GCTs included pazopanib in combination with crizotinib, which showed disease control for four months in one patient, and a PI3K inhibitor which achieved disease control for nine months in another patient. Dasatinib and megestrol were ineffective in two other different patients. Pazopanib has been demonstrated to be active in advanced GCTs and may be considered as a preferable treatment option.

## 1. Introduction

Granular cell tumors (GCT) are an ultra-rare sarcoma subtype [1]. They have neuroectodermal differentiation and were first described by Abrikossof in 1926 [2]. They can be associated with some syndromes such as LEOPARD syndrome, neurofibromatosis, Noonan syndrome, and Watson syndrome. GCTs represent 0.5% of soft tissue sarcomas (STS), have a female predominance [3,4], and are more common in the fourth to sixth decade, although they may appear at any age [3,4,5] The most frequent locations of GCTs, even with variance amongst series, are the tongue and gastro-intestinal tract [4], followed by the thoracic wall and upper extremities, but they may also appear in the extremities [3], head and neck region, breast, genitals, and respiratory tract. GCTs are predominantly superficial tumors arising from the skin, subcutaneous and submucous tissues, being anecdotic in deep muscular compartments.

**Figure 1:**
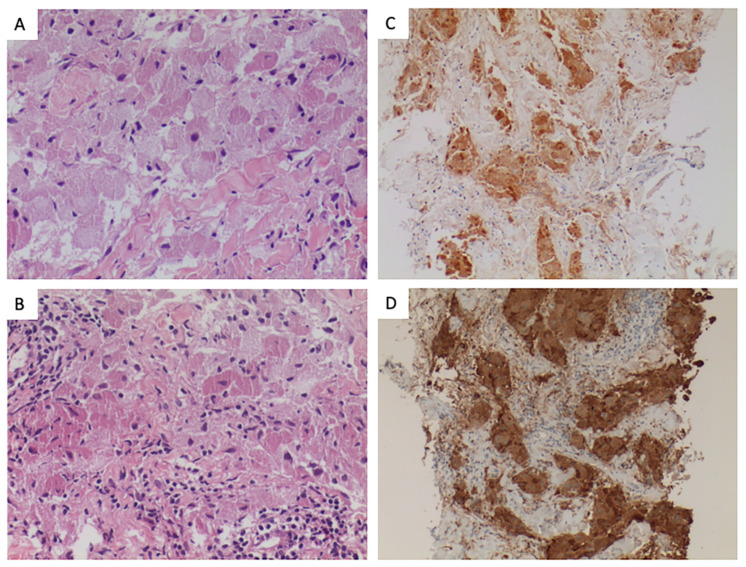
Pathological features of the tumor. (**A**). (H-E, 100×) Granular cell tumor composed of nests of large epithelioid cells, with indistinct cell borders, having abundant granular cytoplasm and small uniform nuclei lacking significant pleomorphism. (**B**). (H-E, 100×). Perivascular moderate lymphocytic inflammatory infiltration was present. (**C**). (S100, 100×). Neural differentiation was present, as expressed by the S-100 staining. (**D**). (CD68, 100×). Tumoral cells express CD68, marker associated with lysosomes and granular change.

GCTs histologically present nests of cells with abundant eosinophilic granular cytoplasm corresponding to lysosomal inclusions. Immunohistochemistry is positive for S-100 protein and vimentin and other markers, like NK1-C3 and CD68, are also commonly expressed [5,6]. (Figure 1) More than 70% of GCT harbor loss-of-function mutations in V-ATPase accessory genes, ATP6AP1 and ATP6AP2. These alterations have been described exclusively in this entity and seem to be pathognomonic for this diagnosis. These genes are involved in endosomal pH regulation and lead to the characteristic intracellular accumulation of granules identified in GCT [1,7,8].

Two main pathology scales have been proposed to distinguish malignant from benign GCTs: Nasser’s and Fanburg’s [5,9]. Fanburg’s is the most widely used scale and its criteria include: necrosis, pleomorphism, a high mitotic index, spindle cells, large nucleoli, and high nuclear/cytoplasmic ratio [5]. However, this pathological classification does not reliably correlate with biological behavior, since some pathologically benign lesions will develop metastases [3,5,10,11].

Overall, among all GCTs, 2–12% can develop regional or distant metastasis, usually to the lungs and bones [5,10]. Surgery is the mainstay treatment of resectable GCT [3,12]. Metastatic GCTs have an aggressive behavior and patients have a poor prognosis, with an estimated median overall survival of 10 months. This life expectancy is worse compared to the whole group of STS [10]. Management of unresectable/metastatic GCTs is challenging, as this is a relatively chemo-resistant entity [3] and, therefore, research on the effectiveness of novel drugs is a priority.

The use of new therapeutic targets on certain STS has been possible thanks to novel knowledge about the biology of such tumors. This is the case of ALK inhibitors such as crizotinib for inflammatory myofibroblastic tumors [13], antiangiogenics for the treatment of alveolar soft-part sarcoma (ASPS) [14,15], crizotinib for clear-cell sarcomas [16], CSF-1R inhibitors in tenosynovial giant cell tumors [17,18] or mTOR inhibitors in PEComa [19]. Similarly, novel advances regarding the molecular biology of GCTs may contribute to finding effective drugs for the treatment of this rare disease.

Some cases reported in the literature have described the use of targeted therapies in malignant GCTs. In particular, there is growing evidence coming from case-reports on the use of pazopanib [20,21,22,23,24,25]. However, currently, there are no recommendations about the preferred systemic therapy for metastatic GCTs. A comprehensive review of all the reported cases might help with clinical decision-making.

The rationale for the use of pazopanib, a multi-tyrosine kinase inhibitor, in malignant GCTs was mainly based on the extrapolation of the data from the PALLETE trial—a phase III study that assessed the efficacy of pazopanib vs. placebo in pretreated STS patients [26], and on the finding of mutations in the angiogenesis pathways in the context of GCTs [21]. Nevertheless, despite the fact that PALETTE proved the effectiveness of pazopanib in STS (with the exception of liposarcomas), malignant GCTs were barely represented in this study. On the other hand, in spite of the finding of mutations of specific genes involved in angiogenesis pathways in isolated cases of malignant GCTs, such as VEGF-A [21], currently there are no validated molecular predictive biomarkers described for pazopanib efficacy.

## 2. Histopathology

Granular cell tumors are characterized by infiltrative, non-encapsulated nests, cords, or sheets of polygonal and occasionally spindled cells with uniform nuclei, abundant eosinophilic, finely granular cytoplasm, without a high nuclear to cytoplasmic ratio [1,4,6]. Many of the tumors have Pustulo-ovoid bodies of Milian, which are large eosinophilic granules with clear halos. The granules are periodic acid-Schiff (PAS) positive and diastase resistant, and they represent lysosomes [1,4,6,27]. The nests are often separated by fibrous tissue and located in the dermis, subcutis, or submucosa. Nuclei have dense chromatin, are relatively small, and are centrally placed. Prominent lymphocytic infiltrates are identified in 35% of GCTs [4,28].

These lesions can be sub-classified into benign, atypical, and malignant categories based on histologic features. The first system was developed in 1998 by Fanburg-Smith et al. using these six features: necrosis, increased mitotic count (greater than two per ten high-power fields), spindled tumor cells, nuclear pleomorphism, vesicular nuclei with prominent nucleoli, and high nuclear-to-cytoplasmic ratio [5]. Lesions with none of these features are categorized as benign, and those with one or two features are categorized as atypical. Tumors with three or more are called malignant and have a considerably worse prognosis. Nevertheless, the pathology classification is not predictive enough of behavior.

We must remember that benign tumors can also demonstrate both vascular and perineural invasion, but these histologic features do not confer malignancy or an adverse prognosis [5,6].

Both benign and malignant tumor cells typically stain positively for S-100, CD68, neuron-specific enolase, CD57, inhibin, calretinin, TFE3, SOX10, and nestin [6,29,30,31].

The Ki-67 index has been described as a useful tool for differentiating between GCT subtypes: Ki-67 of less than 5% in benign GCT, 5–10% in atypical tumors, and 10–50% in many malignant GCTs. Cytokeratin, epithelial membrane antigen, myogenic (desmin, myogenin, smooth muscle actin), or melanic markers (Melan-A, HMB-45) are consistently negative in GCTs [5].

Although extremely rare, GCT variants that do not express S100 protein have been described and named non-neural GCT. They show reactivity for CD68, NKIC3, CD10, and α1-antitrypsin, and expression of neuron-specific enolase and PGP9.5 has been reported [5,32].

## 3. Molecular Biology

The most accepted theory is that upon peripheral nerve injury, S100 protein is released from Schwann cells in damaged nerves. This causes the high concentration of S100 protein frequently detected in GCT with immunohistochemistry. The S100 protein-activation promotes Schwann cells migration during the course of repair of injured nerves. In the presence of tumor cells, the system, cell damage-activated Schwann cell-S100 protein secretion reinforces the instability, causing cell proliferation and tumor growth [33].

By whole-exome sequencing and targeted sequencing analysis, recurrent ATP6AP1, *ATP6AP2*, *ATP6V0C* inactivating somatic mutations have been identified in up to 72% of the GCTs. Subsequent in-vitro impairment of these pH regulators in Schwann cells not only spurred oncogenesis via increased phosphorylation of PDGFR-B, SFK, and STAT-5, but it also resulted in impaired vesicle acidification, impaired endocytosis, and the build-up of intracytoplasmic granules. It is also possible that MITF/TFE3 nuclear localization and activation due to V-ATPase dysfunction contributes to GCT tumorigenesis, since TFE3 gene fusions and nuclear localization of the resulting fusion proteins are observed in several types of sarcomas. These mutations are found in less than 0.1% of other cancer types and that is why they are considered pathognomonic. Impairment of ATP6AP1, ATP6AP2, and ATPV0C resulted in impairment of the V-ATPase (H+ ATPase) complex with a subsequent decrease in lysosomal activity. This not only correlates with the characteristic cytoplasmic findings, but it also explains the tumor’s positive immunohistochemical staining for TFE3 and wild-type MITF, as lysosomal inhibition induces activation of transcription factors MITF, TFE3, and TFEB [7,8].

Mutations in genes involving the TGFβ and MAPK pathways have also been described. Mutations in *TGFBR1*, *TGFBR2*, and *LTBP2* eliciting loss-of-function were found and, as a consequence, cells may be able to escape the inhibitory effect of TGF-β leading to increased proliferation. MAP3K15 loss of-function mutation was also described, which is predicted to promote tumor cell survival by inhibiting cell apoptosis. Both pathways are known to play essential roles in the regulation of the cell cycle, tumor formation, and metastasis of many cancers. While they usually operate in opposing roles, with TGFβ-suppressing cell proliferation and MAPK-promoting proliferation, in the event of malignant transformation, both seem to support each other and promote growth and metastasis with TGFβ switching from tumor suppressor to a pro-metastatic factor [34].

The PIK3CA gene encodes the catalytic subunit of phosphatidylinositol-3 kinase (PI3K). A variety of tyrosine kinase receptors such as EGFR, ERBB2 (HER2), RET, MET, and VEGFR activates PI3K. PI3K then triggers intracellular downstream AKT/mTOR signaling that promotes cell survival, proliferation, growth, and motility. Malignant GCTs showed alterations in key known driver oncogenes such as *TP53* and *PIK3CA*, while no alterations were found in these genes in benign GCTs [35].

GCTs have also been shown to express CD63, LC3 (microtubule-associated protein 1 light chain 3, a specific marker of autophagy), and the antigen presenting cell marker HLA-DR. HLA-DR and CD68 strong immunoreactivity suggests an antigen-presenting cell (APC) phenotype for GCTs. HLA-DR expression is associated with APCs (monocytes, macrophages and dendritic cells), as well as B lymphocytes, and activated T lymphocytes. The significance of these findings is still under debate, and it is possible that, in addition to CD68 immunoreactivity, HLA-DR immunoreactivity might be reactive in nature and have a role in antigen presentation during the innate immune response in GCTs. It is also plausible that HLA-DR expression might be a positive prognostic factor for survival. Another possibility is that the presence of HLA-DR immunoreactivity might suggest an epithelial–mesenchymal transformation, more indicative of tissue dedifferentiation than an involvement in tumor antigen presentation [36].

Understanding the role of these alterations is critical for the research and development of systemic treatments.

## 4. Review of the Literature of Systemic Teraphy

### Methods

**Figure 2:**
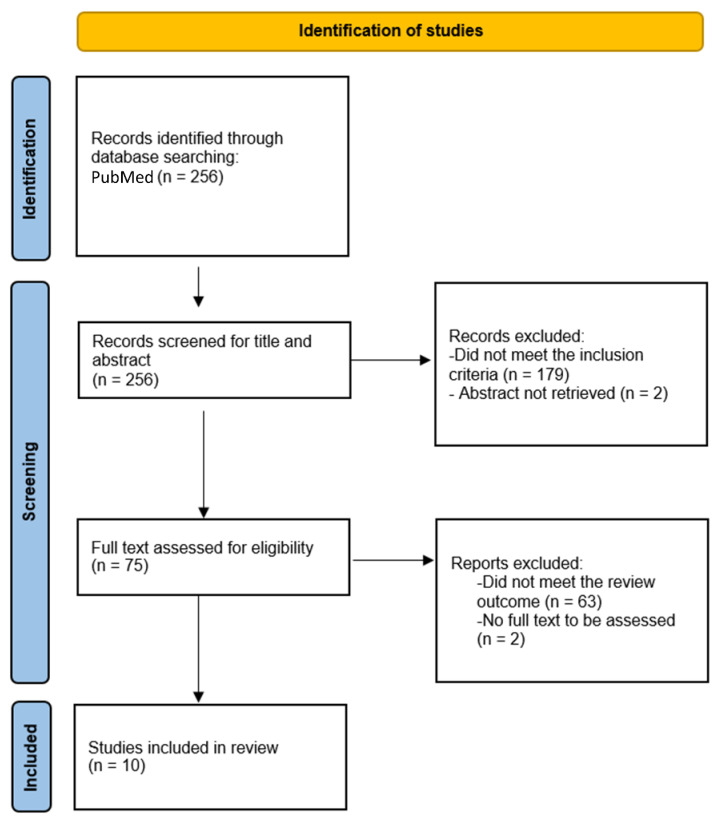
Flowchart showing selection of articles for the present review of the literature.

We performed a review of the available literature with regards to patients with unresectable and/or malignant granular cell tumors treated with systemic therapies, excluding chemotherapy. We searched in PubMed using the terms: “malignant granular cell tumor” OR (“granular cell tumor” AND (“metastatic” OR “metastasis” OR “advanced”)). Articles were included in our review if all the following inclusion criteria were met: (1) pathological diagnosis of granular cell tumor-including typical, atypical, and malignant histology; (2) treatment with systemic therapies, excluding chemotherapy; and (3) unresectable and/or metastatic disease at the time of the start of treatment. Exclusion criteria included: full text in a language different from English, French, or Spanish. The study was registered at the Research registry (number 1718). Firstly, articles were selected by title and/or abstract, and then full-text was reviewed among those selected papers (Figure 2).

## 5. Results

The PubMed search resulted in 256 articles. Amongst these, 75 articles that fulfilled the above criteria according to their abstract or tittle were identified. After reviewing the full texts of these 75 articles, 10 articles were finally selected (Figure 2) [20,21,22,23,24,25,37,38,39,40]. The most common target systemic therapy used in GCTs according to this review of the literature was pazopanib, (Table 1).

Data on the efficacy of other systemic therapies were also reported in case-reports. Morita et al. reported a case of malignant GCT treated with a PI3K inhibitor in the context of a clinical trial. The patient experienced disease control with stable disease for nine months [22]. Other systemic therapies, such as dasatinib or megestrol, did not show satisfactory results, with both patients experiencing rapid disease progression [21,39]. Pazopanib in combination with crizotinib was also reported in one patient, with disease control lasting four months [37].

Characteristics of the cases treated with pazopanib are summarized in Table 1. This sarcoma subtype exhibits a marked ubiquity with primary sites reported more frequently in trunk-wall, head-and-neck, and extremities, in this collected series. All but one patient had distant metastasis, with the lungs being the most frequent site, followed by bone and soft-tissue. One of the patients had locally advanced unresectable disease. Five patients had received previous systemic therapy, with limited benefit in terms of progression-free survival while no objective responses were detected. Previous treatments were as follows: three patients received chemotherapy (the different schemes administered were carboplatin/paclitaxel + cetuximab, gemcitabine/docetaxel, doxorubicin/ifosfamide) [23,24,38] and no responses were reported, one patient received dasatinib progressing after two months, and one patient received smoothened inhibitor, after progression a PI3K inhibitor was administered with disease control lasting nine months [21].

**Figure 3:**
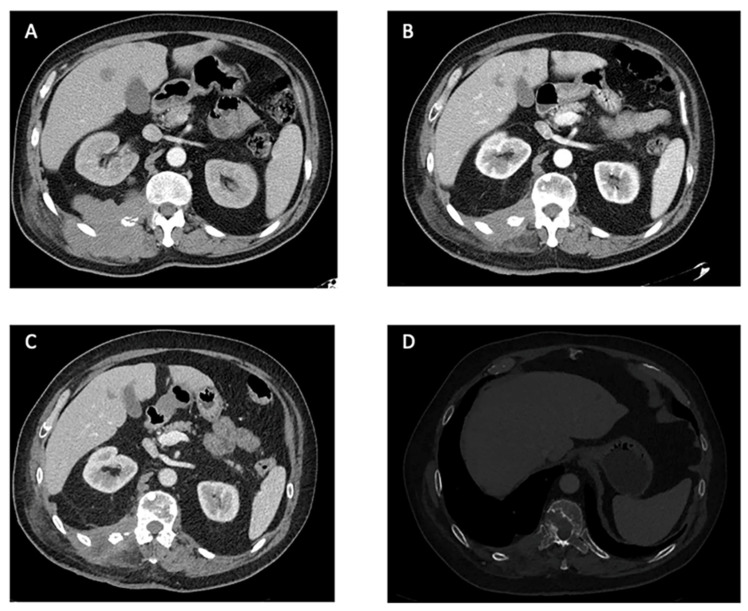
Computer Tomography Scans of a patient treated with pazopanib at our center: an example of PR. (**A**). Locally advanced disease affecting the right paravertebral area with involvement of the right 12th vertebral transverse process and rib and metastatic liver nodes. Baseline for the treatment with pazopanib. (**B**). RECIST 1.1 partial response of the mass in the right paravertebral area after 20 months under treatment with pazopanib. (**C**). Maintained partial response on primary tumor and hepatic lesions after 25 months under pazopanib. (**D**). Increase in size of a bone lesion in D10 establishing mild spinal cord stenosis, 25 months after the start of pazopanib (bone window).

The median reported time of response duration with pazopanib was available for nine patients and was seven months. However, five patients were still on therapy at the time of the report. Eight of the patients experienced disease control with pazopanib, with four patients achieving an objective response whereas two patients experienced progression disease as the best response (Figure 3).

Molecular analysis based on next generation techniques was performed in three out of ten cases. One of the patients exhibited overexpression of sixteen genes including *SRC*, *MET*, and *VEGFA* [21]. Another case showed a somatic mutation in the additional sex comb-like 1 (ASXL1) gene [22]. In the third analyzed case, loss-of-function mutation in BRD7 and a mutation in GFRA2 were identified [23]. No other genomic alterations were reported.

## 6. Discussion

To the best of our knowledge, this is the first ever reported review of patients with advanced GCTs treated with targeted therapies with a special focus on pazopanib.

Metastatic GCT represents a very small fraction; 1–2%, of all the GCTs [1] and is associated with poor survival [10]. Of note, one case did not fully accomplish the pathological criteria defined for malignancy and still developed distant metastasis [23]. This reflects the difficulty in anticipating the clinical behavior of GCT at a pathology level. Probably, using the term “malignant” as a counterpart to a benign GCT condition should be avoided in a similar way to that which occurred in solitary fibrous tumors or gastrointestinal stromal tumors; where it was deemed more rational to talk about metastatic risk rather than the polarized terms of benign or malignant GCT.

Tongue and gastro-intestinal, followed by trunk-wall, are the most common locations for primary GCTs reported in the literature. However, trunk-wall, limbs, and head-and-neck were the most frequent in our series with three cases each. We cannot affirm that some locations are more prone to develop metastasis within the ubiquitous GCTs, but in uncommon locations such as head-and-neck could be a hypothesis. As a matter of fact, GCTs are predominantly superficial tumors [2,3,4]. By contrast, in our collected cases, we could deduce five were deep tumors and five lacked this information at diagnostic time. This suggests that GCTs with deep presentation might be more aggressive than superficial ones, and it could be an interesting focus of study for larger series in the future. Since no prospective data on systemic therapy is available for GCTs, this remains an unmet need for clinical decision-making. Neither do we have any retrospective series, but just isolated case-reports [20,21,22,23,24,25,37,38,39,40]. The pivotal randomized phase III trial that positioned pazopanib as an active treatment for second lines in advanced STS, PALETTE, enrolled patients with different histologies but provided hardly any anecdotal experience for uncommon STS subtypes [26]. Furthermore, even when some clinical trials have addressed, at a multinational level, the efficacy of pazopanib or other tyrosine kinase inhibitors (TKI) in uncommon STS sarcomas [15,41,42,43], none of them have been conducted in GCT. It is probable that a worldwide global collaborative effort would be needed for this aim. Thus, the analysis of data from case-reports constitutes a valuable exercise in an ultra-rare entity such as that represented by advanced GCT.

The efficacy of classic cytotoxic chemotherapy in clinically malignant GCTs seems very limited, based on the published evidence [3,38], with only two cases reported in the literature achieving a notorious dimensional response. One of these cases was treated with two cycles of gemcitabine plus paclitaxel [6] and the other case responded to six cycles of carboplatin plus etoposide [44]. Notably, three cases reported in this review received chemotherapy prior to pazopanib, obtaining no objective response.

In the pivotal trial, Pazopanib, approved in advanced non-selected STS, except for liposarcomas, for second or further lines [26,45], exhibited an overall response rate (ORR) of 6%, with the most relevant added-value being the median of progression-free survival (4.6 months vs. 1.6 months compared with placebo). In marked contrast, the ORR of the GCT cases pooled in this review that were assessable for response was 40%. This outcome represents the greatest activity in terms of response rate for pazopanib in any STS subtype. With respect to the duration of disease control, it is more difficult to compare since this data is not easily drawn from these case-reports. Additionally, due to the variability in behavior, the median of survival reported in the literature widely ranged from 44 months [45] to 10 months when the analysis focused mostly on metastatic disease [38].

Mutations in vacuolar ATPase (V-ATPase) complex have been identified in more than 72% of GCTs [7,8]. Functional studies suggest that these loss-of-function mutations could have oncogenic properties in GCTs, as they lead to an increase in phosphorylation and consequently enhanced signaling through several molecular pathways, such as SFKs, STAT5a/b, PDGFR-β and the activation of the Wnt pathway [7,8]. None of the analyzed cases reported the V-ATPase status, so we can only hypothesize on this point. Other isolated information derived from sequencing in GCTs has identified alterations in VEGFA [21], TERT promoter, in the PI3K pathway [46], and in the MAP-kinase pathway [46,47,48,49,50,51].

The mechanisms responsible for the activity of pazopanib in malignant GCTs could then rely on its inhibition capacity on several of the overexpressed pathways in this entity, such as Src and PDGFR-β. Other identified genetic alterations in GCTs [21,23] could also be directly (MET) or indirectly (BRD7 through RET) inhibited by pazopanib [23,52,53,54].

Considering the kinase inhibitory profile of pazopanib, PDGFR-β represents one of the most favorable kinase inhibitory targets for pazopanib and, even when it was not found to be overexpressed in aggressive GCTs compared with less aggressive GCTs, it cannot be ruled out as a potential driver. However, some CGT were shown to be not sensitive to dasatinib, which is also a PDGFR-β inhibitor [21]. Indeed, this latter TKI is a powerful inhibitor of Src, and this could indicate that it does not have a prominent driver role in GCTs. Thus, further investigations are required to identify the driving signals behind the response to pazopanib in GCTs. In our case, it was not possible to obtain adequate tumor tissue, due to secondary changes observed after pazopanib that precluded it from undergoing sequencing analysis.

A potential strategy to increase the efficacy of pazopanib, already being tested in other STS and which could be useful for GCTs, is the combination of pazopanib with other agents such as trametinib—an inhibitor of the RAS-MAP kinase pathway [55], or abexinostat—a histone deacetylase inhibitor [56]. Some preclinical data support the hypothesis that these drugs could synergize with pazopanib in the context of GCTs. On the one hand, the fact that GCTs can be associated with Noonan or neurofibromatosis syndromes [48,49,50,51,57] could emphasize the relevance of RAS-MAP kinase pathway genes also in GCTs [47]. Additionally, the reported mutations in the *BRD7* gene [23,58] or the positivity of phosphorylated histone H3 found by immunohistochemistry in GCTs [35,59] could suggest alterations in the histone acetylation mechanisms.

Furthermore, the inhibition of the PIK3CA pathway has been suggested as potentially useful in “malignant” GCTs. Whole exome sequencing detected alterations in the *PIK3CA* gene, which were not present in the “benign” GCTs [35]. In line with this, one patient in this compilation achieved stable disease for 9 months with a PIK3 inhibitor [22].

Prominent lymphocytic infiltrates are identified in 35% of GCTs [4,28]. This finding could support the exploration of PD1 inhibitors in this entity. Combination therapy with immune checkpoint inhibitors and antiangiogenics have been tested in selected advanced STS [60,61], with apparent higher efficacy with respect to monotherapy by indirect comparison. Alveolar soft part sarcomas (ASPS) have demonstrated special sensitivity to this therapeutic approach. Interestingly, Transcription Factor E3 (TFE3) is consistently overexpressed in ASPS due to the specific recurrent driver gene translocation ASPS-TFE. Curiously, TFE3 is also overexpressed in 53–91% of GCTs [62,63]. Some authors speculate that TFE3 could be related to immune-sensitivity through the direct effect on T-lymphocytes [64].

There is little reference to toxicity of different treatments used in GCTs. There is no reference to adverse events in any of the chemotherapy cases, Megestrol, or PI3K inhibitor [21,22,23,24,38]. The period of exposure to chemotherapy and Megestrol was of short duration due to rapid progression, but there were no specifications [38]. When Dasatinib was used, grade 1 toxicity was reported but the specific symptoms were not specified [21]. In one case, Pazopanib was suspended due to grade 3 fatigue [22]; there were also reports of grade 2 such as hypertension and one case of diarrhea without grade specification [21,22]. In Pazopanib cases, the toxicity reports were within the expected according to the findings of the clinical trials in other soft tissue sarcomas [26].

In one of the reports, Sunitinib was used as a second line treatment after Pazopanib failure with no response registered and rapid progression, and no adverse effects were reported [23].

In our center, we treated one patient with the characteristics of these reviews, a 52-years old male with a lesion arising in the right paravertebral muscles, being superficial at diagnosis. After resection of the primary tumor, a local, lymphatic, and hepatic relapse occurred 8 years later. The initial treatment following the recurrence was chemotherapy, based on ifosfamide 1800 mg/m^2^ days 1–5 and etoposide 100 mg/m^2^ days 1–5. A total of eight courses were administered with good tolerance and stable disease as it best response. After a new progression, the patient received 25 months of pazopanib with good tolerance. Partial response was achieved according to RECIST criteria (Figure 3) and discontinuation was due to progression. The duration of treatment with pazopanib and the survival time was longer in our case than in cases reported in this review, while the results of chemotherapy were similar.

Limitations of this review are inherent with the retrospective nature of case-reports. This conditioned the lack of further relevant information such as progression free-survival or a more homogeneous radiological assessment, or follow-up schedule. Despite these limitations, we believe that this piece of evidence is valuable as it summarizes the current available data of pazopanib activity and other targeted therapies in this ultra-rare sarcoma.

## 7. Conclusions

GCTs are extremely rare sarcomas, they have clearly identifiable pathological characteristics, but the current subtyping into benign, atypical, and malignant, although useful, is not a clear predictor of metastatic capacity or the level of clinical aggressiveness that the tumor will present. Larger series are needed to analyze a potential correlation between deep location and higher aggressive behavior, compared to superficial presentation in GCTs. Advances in the molecular biology of this type of sarcoma have detected pathognomonic mutations that, although not fully understood, can be useful for research of therapeutic lines.

The literature compilation shows that pazopanib demonstrates substantial activity in terms of dimensional responses that could eventually translate into a longer survival in advanced GCTs. The exact underlying mechanisms for the notorious efficacy of pazopanib are not yet fully elucidated, although it could be related to loss-of-function of *ATP6AP1/2* genes. Inclusion in clinical trials that target potential drivers in GCTs should always be considered.

## 8. Future Directions

Further investigation into the pathogenesis of this sarcoma subtype continues. Alterations in the MAPK [46,51], PI3K [46], TGFβ [34] pathways, and the description of VATPase [7,8] variants are considered promising therapeutic targets that may provide novelties in the near future. At the same time, immunotherapy is a field that has not yet been explored and, as we previously mentioned, we have preclinical elements that would support research in this area [60,61]. At the time of this publication, there are no records of ongoing clinical trials in this type of pathology. The low frequency of this entity, especially if we focus on locally advanced or metastatic spread context, will force for a global collaborative effort for achieving new relevant insights in the knowledge of this disease.

## Figures and Tables

**Table 1:** Patient and therapy characteristics from the available published cases of patients with malignant GCT treated with pazopanib.

Reference	Primary Site	Metastases	Previous Systemic Therapies	Line	Duration of Treatment	Best Response (RECIST)	Cause of Withdrawal	Genes Involved
McGuire, 2004 [20]	Upper back	Lung, soft tissues	None	First	>5 months	SD	*	-
Conley, 2014 [21]	Peri-scapular	Lungs	Dasatinib	Second	>4 months	PR	*	*SRC*; *MET*; *VEGFA*
Tan, 2014 [37]	Foot	Bone	None	** First	4 months	-	PD	-
Morita, 2015 [22]	Orbicular	Lungs	Smoothened inhibitor, PI3K inhibitor	Third	7 months	PR	Toxicity (fatigue)	*ASXL1*
Wei, 2015 [23]	Pharyngeal	Bones, soft tissues	Gemcitabine plus docetaxel	Second	6 months	PR	PD	*BRD7*; *GFRA2*
Imanishi, 2016 (Case 1) [38]	Elbow	Lung, lymph nodes	None	First	-	-	PD	-
Imanishi 2016 (Case 2) [38]	C5 root	Lung, lymph nodes	Adriamycin plus Ifosfamide	Second	>12 months	SD	-	-
Katiyar, 2020 [24]	Lower lip	^#^ None	Carboplatin plus paclitaxel and cetuximab	Second	10 months	PR	^&^ Death	-
Tian, 2018 [25]	Posterior calf	Lung, colon	None	First	^∆^ 48 months	SD	*	-
Karasavvidou, 2023 [40]	Urethra	Lung, pelvis	None	First	10 months	SD	*	-

PR: Partial response. SD: stable disease. PD: Progressive disease. * Still on treatment with pazopanib at the time of publication. ** This patient received pazopanib in combination with crizotinib as a first line. ^#^ Locally advanced unresectable, with invasion of locoregional structures including central nervous system. ^&^ Sudden death without suspicion of PD; ^∆^ Isolated limb infusion with chemotherapy and finally, transfemoral amputation was also performed for local control while in therapy with pazopanib.

## Data Availability

Not applicable.
